# How Healthy Lifestyle Habits Have Interacted with SARS-CoV-2 Infection and the Effectiveness of COVID-19 Vaccinations: A Comment

**DOI:** 10.31662/jmaj.2024-0216

**Published:** 2024-11-01

**Authors:** Hinpetch Daungsupawong, Viroj Wiwanitkit

**Affiliations:** 1 Private Academic Consultant, Phonhong, Lao People’s Democratic Republic; 2 Saveetha Medical College and Hospital, Saveetha Institute of Medical and Technical Sciences, Saveetha University, Chennai, Tamil Nadu, India

**Keywords:** lifestyle, habit, SARS-Co-V2, vaccination

## Dear Editor,

We would like to comment on “How Healthy Lifestyle Habits Have Interacted with SARS-CoV-2 Infection and the Effectiveness of COVID-19 Vaccinations: Tohoku Medical Megabank Project Birth and Three-Generation Cohort Study ^(1)^.” This study presents critical findings on the relationship between vaccination and SARS-CoV-2 infection, emphasizing how lifestyle factors can interact with vaccine efficacy over time. Data obtained from 2014 to 2019 provide a thorough picture of the lifestyle of participants before the pandemic. The study used robust techniques, such as Cox proportional hazards models and logistic regression, and found that those who received five doses of vaccine had a considerably lower risk of SARS-CoV-2 infection, regardless of lifestyle. Vaccinated persons who reported higher sleep satisfaction had a larger reduction in risk, implying that lifestyle factors may influence vaccine efficacy.

However, one weakness of this study is its dependence on self-reported lifestyle variables, which may be subject to bias or imprecision, thereby affecting the reliability of conclusions drawn concerning the relationship between sleep satisfaction and SARS-CoV-2 vaccination. Furthermore, the study did not account for other potentially confounding characteristics, such as socioeconomic status and access to health care, which could influence lifestyle habits and vaccination rates. This omission may obfuscate nuanced interactions between lifestyle variables and vaccine outcomes, leading to an oversimplified interpretation of the study results.

Future studies should try to gain a better understanding of how lifestyle factors affect vaccine efficacy, especially as the pandemic progresses and new mutations appear. This approach could involve long-term studies that account for a broader range of population parameters and objectively measure lifestyle variables, such as wearable sleep quality assessments rather than self-reports. Furthermore, studying the long-term efficacy of immunization in conjunction with other lifestyle treatments could provide valuable information for optimizing public health efforts to battle diseases like COVID-19 in various populations.

In terms of innovation and improvement, future research might look into the processes underlying the observed relationship between sleep satisfaction and vaccine efficacy. Examining immune response indicators in conjunction with vaccination and lifestyle factors may help to understand the molecular basis of these associations. Furthermore, programs that promote vaccination and healthy lifestyle choices should be evaluated for their synergy. Such research would not only broaden our understanding of vaccine interactions but would also help to drive public health measures by advocating for a comprehensive pandemic response strategy.

## Article Information

### Conflicts of Interest

None

### Author Contributions

HP 50% ideas, writing, analyzing, approval

VW 50% ideas, supervision, approval

### Data Availability

There is no new data generated.

### AI Declaration

The authors used a language editing computational tool in preparing the article.

### Approval by Institutional Review Board (IRB)

Not applicable
